# Cross Sectional Study on the Association between Dental Caries and Life Habits in School Age Italian Children

**DOI:** 10.3390/healthcare10040607

**Published:** 2022-03-24

**Authors:** Massimiliano Ciribè, Angela Galeotti, Chiara Dolci, Livia Gargiullo, Martina Mammone, Erika Cirillo, Paola Festa, Giuseppe La Torre

**Affiliations:** 1 Dentistry Unit, Bambino Gesù Children’s Hospital, IRCCS, Viale Ferdinando Baldelli 41, 00146 Rome, Italy; massimiliano.ciribe@opbg.net (M.C.); chiara.dolci@opbg.net (C.D.); martina.mammone@opbg.net (M.M.); erika.cirillo@opbg.net (E.C.); paolafesta1@gmail.com (P.F.); 2 Pediatric Unit, Bambino Gesù Children’s Hospital, IRCCS, Piazza Sant’Onofrio 4, 00165 Rome, Italy; livia.gargiullo@opbg.net; 3 Department of Public Health and Infectious Diseases, Sapienza University of Rome, Piazzale Aldo Moro 5, 00185 Rome, Italy; giuseppe.latorre@uniroma1.it

**Keywords:** dental caries, caries risk, epidemiology, school age population, sweetening agent

## Abstract

Dental caries is still a major public health issue and influences the overall health of children. The risk factors for caries include biological, socio-behavioral, and environmental factors. Our aim is to assess the association between dental caries and the life habits of children and their parents. A cross-sectional study was conducted in Rome (Italy) among primary school children aged 5 to 11. Parents completed the anamnestic questionnaire, and a dental clinical examination was performed on 333 children. Caries prevalence was 38.7% overall, 47% in males and 31.9% in females. The association between bottle night-time feeding and caries was statistically significant (43.2%; *p* = 0.013). Usage of a honeyed pacifier was also significantly associated with the presence of caries (72.7%; *p* = 0.027). Finally, higher caries prevalence was found among male children (47% vs. 31.9%; *p* = 0.005). The present study shows that the percentage of caries is still high in the paediatric population, and caries prevalence is associated with life habits. Our results highlight the importance of oral health education programs at primary school that involve teachers and parents to contribute to improving lifestyles.

## 1. Keynotes

Dental caries is still a major public health issue. This study aims to assess the association between dental caries and the life habits of children and their parents. The analysis of the parameters highlights a statistically relevant association between caries and the following variables: bottle night-time feeding, use of a honeyed pacifier, and male sex.

## 2. Introduction

In recent years, Western countries experienced a significant reduction in the onset of dental caries due to oral health education promoted by the scientific community, and evidence-based prevention measures [1]. Nevertheless, dental caries is still a major public health issue in many countries. According to recent evidence reported by the World Health Organization (WHO), 21.6% of children aged 4 and 43.1% aged 12 suffer from dental caries in Italy [1,2]. Italian national guidelines highlighted the importance of undergoing a first dental visit at the age of 18–24 months so that any oral disease can be diagnosed early [1]. Many studies widely show that dental caries prevention is essentially based on daily dietary and oral hygiene practices [1,3,4,5,6]. Parental oral health behaviors may have a significant impact on the development of their children’s oral hygiene habits and, consequently, on the prevalence of oral diseases, children’s tooth brushing, and dietary habits (high sugar diet, feeding practices, and nocturnal breastfeeding) [4]. Free sugar in the diet, in fact, represents an important risk factor for dental caries [7].

Scientific evidence shows that promoting oral health in the earliest stages of life is the best approach to establish good personal habits, which will be reinforced during adolescence and endure through adulthood [1,2,3].

School is obviously one of the best contexts where to deliver good life habits, so it can be an important meeting point for healthcare professionals and children, their parents, and teachers. Health prevention programs can thus be created to teach children relevant knowledge on oral health and the right oral hygiene maneuvers to perform at home [1,2,3].

Furthermore, school is an important source for carrying out epidemiological surveys and analysing the interaction between students’ oral health and other conditions. 

In line with these statements, the correlation between oral diseases and life habits should be analysed. Although there are many studies that show a correlation between the number of dental caries and life habits, most of these studies show conflicting results, especially about breastfeeding [8,9,10].

The study hypothesis stated that there is a correlation between dental caries and the life habits of children and parents. The aim of this study is to evidence the association between dental caries and children’s life habits as well as those of their parents in children aged 5 to 11.

## 3. Materials and Methods

A cross-sectional study was conducted in Rome (Italy) at Comprehensive Institute “VIRGILIO” between October 2018 and January 2019. The study was approved by the Chief Medical Office of Bambino Gesù Children’s Hospital, IRCCS (Rome). Parents of primary school children were invited to fill out an anamnestic questionnaire aimed to investigate the life, eating, and oral hygiene habits of both parents and children (Supplementary Materials Table S1). In particular, the following parameters were investigated:

- Breastfeeding
- Bottle feeding
- Night-time feeding
- Child allergies
- Child mucolytics or cough suppressants
- Child mouth breathing
- Child systemic fluoride
- Child daily number of brushings
- Fluoride toothpaste
- Child dental flossing
- Pacifier
- Bacterial Colonization
- Honey
- Night-time bottle
- Child sex
- Single mother
- Mother No. of children
- Smoking mother
- Mother caries
- Mother annual dental visits
- Mother daily brushing
- Mother systemic fluoride during pregnancy
- Smoking father
- Father annual dental visits
- Father caries

Parents were asked to sign a release to consent to dental examinations and participation in the study.

All school children were included in the study except those who did not have parental consent. Clinical examination was performed on 333 children. The visits were all carried out by the same operator with 10 years of experience in pediatric dentistry to avoid any divergence of assessment. Dental examination was performed using a WHO periodontal probe under natural light and a standard size-4 mirror, following the WHO recommendations for oral health surveys [11].

The following parameters were recorded during the visit for each examined child:

- Sex
- Age
- Presence/absence of caries
- Number of decayed teeth

## 4. Statistical Analysis

The exposure variables in the current study were social and demographical characteristics (child sex, child age) and the child’s oral behavior (night-time feeding, honey), while the outcome variable was the child’s oral hygiene status (presence of caries). 

The recorded clinical data were summarised in a table and subjected to statistical analysis. Contingency tables were used to carry out the analysis. Each i value of the variable is associated with the ni number of times in which that value occurs in N observations or its relative frequency (ni/N). 

The statistical analysis was performed using the Pearson chi-square test or Fisher exact test, where applicable, to analyse the prevalence of dental caries and their association with all other variables analysed in this study.

The statistical significance level was set at *p* value < 0.05. Dental caries prevalence in children with an average age of 7 years old was expected to be around 22%. Our survey on 338 subjects revealed an estimate of caries prevalence with a ±2% error and a 95% interval of Confidence (CI). 

The statistical analysis was carried out using the statistical software SPSS, release 26.0.

## 5. Results

Clinical examination was performed on 333 (98.5%) children (168 males and 165 females) with an average age of 7.86 years and a standard deviation of 2.4. Clinical examination was performed after obtaining signed informed consent from their parents. 

Two children were excluded from the study because they did not cooperate during the dental visit. Three children were excluded from the study because the questionnaires were not completely filled out by parents. The prevalence of caries was 38.7% overall, 47% in males and 31.9% in females. 

Table 1 shows all the variables with the complete results. The association between bottle night-time feeding with milk or a drink except water and caries was statistically significant (43.2%; *p* = 0.013; 95% CI: 0.147–1.008). Usage of a honeyed pacifier was also significantly associated with caries presence (72.7%; *p* = 0.027; 95% CI: 0.725–14.098). Finally, higher caries prevalence was found among male children (47% vs. 31.9%; *p* = 0.005; 95% CI: 0.365–1.066). 

No statistically significant association was shown between dental caries and all other variables. However, some variables show differences in percentage terms. For example, 46 of the 333 children took systemic fluoride, had a prevalence of caries of 50% compared to 38.3% of children who did not take systemic fluoride. On the other hand, usage of fluoridated toothpaste was associated with caries presence with a prevalence of caries of 37.7% compared to 47.9%. Higher caries prevalence was found among children whose mothers had caries (41.5% vs. 29.4%). 

## 6. Discussion

This study investigated dental caries prevalence in children aged 5 to 11 and assessed the association between dental caries and the life habits of children and parents.

The new guidelines published by WHO in 2015 recommended reducing the intake of free sugars (mono and disaccharides such as glucose, fructose, and sucrose) to less than 10% and of sugars (honey, juice, etc.) to below 5% of total energy intake both in adults and children [12]. Although the use of traditional preventive methods has been successful in reducing dental caries, there is still a need to develop and evaluate new preventive approaches. Caries should be detected and monitored in its early stages when the reversal of the carious process can still occur [13].

Our results showed an association between using a honeyed pacifier and dental caries, as already suggested in previous studies [14,15]. A systematic review showed that breastfeeding beyond the age of 12 months associated with nighttime feeding increases the prevalence of caries [14]. Another systematic review from the literature, carried out by Tham et al. in 2015 and including 63 papers about the risk of caries related to breastfeeding, highlighted that children who were breastfed for more than 12 months had an increased risk of developing caries compared to those who were breastfed for a shorter amount of time (69.3%). Amongst children breastfed for more than 12 months, those who were also fed during the night or more frequently had a further increase in caries risk (77.1%) [15].

As to dental caries prevalence related to sex, our data show a higher prevalence in boys. Our data were not aligned with the information suggested in the literature [16].

Recent papers studied the association between dental caries and sex. A recent study conducted in the United Kingdom showed no gender difference in caries formation in prepubescent age, while from the age of 12 years, an increase was shown in the female population. Studies in the adults confirmed dental caries developed more in the female population, in contrast to the results of this study [17,18].

Our sample did not show statistically relevant associations between dental caries prevalence and allergies or mucolytics, cough suppressants and nasal sprays administered to the subjects. Our results are aligned with the information suggested in previous studies [19,20], even though the literature suggests conflicting data on the matter. Mouth breathing is one of the greatest risk factors highlighted and analysed when studying the development of dental caries. In-depth studies have shown that those patients who breathe through their mouth for different reasons complain of having a dry mouth, especially during their sleep or upon awakening [21,22]. These symptoms result from sleep-related xerostomia, which may reduce local antibacterial effects of saliva or reduce its salivary cleansing action [23], leading consequently to a possible increase in dental caries, erosion and gingival inflammation [24,25,26,27,28,29].

Scientific evidence suggests that sleep-related xerostomia is linked with respiratory disorders, such as asthma. These relationships result from a decrease in salivary production due to circadian rhythms and dry mouth, mainly caused by breathing during sleep [30,31].

Data on the association between asthma and dental caries are conflicting [30]—some studies report a higher frequency in caries [32,33], while others find no association [21,22].

Furthermore, the symptoms of asthma overlap with those of allergic rhinitis [20,32,34].

Numerous studies show that allergic rhinitis does not constitute a risk factor for dental caries [20], even though oral breathing is the main complication for both of them. It is furthermore essential to consider that asthma and allergic rhinitis often coexist [34]. 

No relevant differences related to dental caries prevalence emerged in subjects who took systemic fluoride and those who did not in this study. Substantive evidence shows that fluoride, through different applications and formulas, works to control caries development. However, the actual mechanism of fluoride action is still a subject of debate. The early studies of Dean et al. (1942) [35] and McKay (1952) [36] suggested that fluoride needs to be ingested to act beforehand in dental caries prevention. During that period, various clinical studies showing the systemic pre-eruptive validity of fluoride were conducted [37,38,39]. In the following years, doubt was cast on the exclusively pre-eruptive effect of fluoride. They concluded that fluoride has a post-eruptive effect and that fluoride supplementation starting from birth is unnecessary [40,41,42,43,44]. It should also be considered that fluoride supplementation increases the risk of fluorosis [45,46,47]. The anti-caries effects of fluoride are primarily topical [48]. Since the fluoride benefit is mainly topical, perhaps it is better to deliver the fluoride directly to the tooth instead of ingesting it [49,50].

No evidence of significant differences between breastfed and bottle-fed children is shown, but night-time bottle feeding with milk, milk and biscuits, milk and honey or camomile emerged as a significant risk factor. This result may be associated with a higher quantity of sugar in bottle content and with prolonged habit [51].

Results also show interesting data with respect to dental caries association in parents and their children. Our results showed a trend of association between dental caries in mothers and the presence of decayed teeth in children, even if not statistically significant. These results may be hypothetically associated with several factors such as familiarity and oral hygiene habits. Two studies confirmed an important relationship between mothers’ oral health and their children’s [52,53]. 

The main limitations of our study are that the subjects’ study comes from only one school in the same city, even if in different locations throughout the city of Rome, the socio-economic status is quite homogeneous in the sample, and the sample size is small. However, the prevalence of caries was 57.1% in children whose parents have a middle school education level. Prevalence is reduced to 34.3% in children whose parents have university degrees. 

Some variables could be statistically significant by increasing the sample size. Therefore, it would be interesting to investigate a larger sample in future research.

## 7. Conclusions

The present survey shows that the percentage of caries is still high in the paediatric population, and caries prevalence is associated with life habits. Our results highlight the importance of oral health education programs at primary school that involve teachers and parents to contribute to improving lifestyles. Since paediatric dentistry plays an important role in oral health care prevention, children should undergo a first dental visit within 24 months of life. 

## Figures and Tables

**Table 1:** Presence of caries compared with the variables analyzed and *p* value.

Variables		Caries		*p*
		NO	YES	
Breastfeeding	NO	20 (55.6%)	16 (44.4%)	0.551
	YES	164 (60.7%)	106 (39.3%)	
Bottle feeding	NO	72 (54.5%)	60 (45.5%)	0.106
	YES	109 (63.7%)	62 (36.3%)	
Night-time feeding	NO	119 (57.8%)	87 (42.2%)	0.179
	YES	62 (66%)	32 (34%)	
Child allergies	NO	154 (60.9%)	99 (39.1%)	0.365
	YES	27 (54%)	23 (46%)	
Child mucolytics or cough suppressants	NO	124 (59.3%)	85 (40.7%)	0.994
	YES	57 (59.4%)	39 (40.6%)	
Child mouth breathing	NO	143 (58.6%)	101 (41.4%)	0.448
	YES	39 (63.9%)	22 (36.1%)	
Child systemic fluoride	NO	156 (61.7%)	97 (38.3%)	0.138
	YES	23 (50.5%)	23 (50%)	
Child daily number of brushing	0	0 (0%)	1 (100%)	0.276
	1	28 (65.1%)	15 (34.9%)	
	2	133 (58.1%)	96 (41.9%)	
	3	23 (69.7%)	10 (30.3%)	
	4	0 (0%)	1 (100%)	
Fluoride toothpaste	NO	38 (52.1%)	35 (47.9%)	0.119
	YES	147 (62.3%)	89 (37.7%)	
Child dental flossing	NO	178 (60.8%)	115 (39.2%)	0.177
	YES	7 (43.8%)	9 (56.3%)	
Pacifier	NO	73 (56.6%)	56 (43.4%)	0.29
	YES	112 (62.6%)	67 (37.4%)	
Bacterial colonization	NO	124 (61.4%)	78 (38.6%)	0.489
	YES	59 (57.3%)	44 (42.7%)	
Honey	NO	176 (60.7%)	114 (39.3%)	0.027 *
	YES	3 (27.3%)	8 (72.7%)	
Night-time bottle	NO	147 (56.8%)	112 (43.2%)	0.013 *
	YES	31 (77.5%)	9 (22.5%)	
Child sex	F	109 (68.1%)	51 (31.9%)	0.005 *
	M	88 (53%)	78 (47%)	
Single mother	NO	181 (60.5%)	118 (39.5%)	0.878
	YES	7 (58.3%)	5 (41.7%)	
Mother No. of children	1	49 (58.3%)	35 (41.7%)	0.199
	2	94 (64.8%)	51 (35.2%)	
	3	34 (56.7%)	26 (43.3%)	
	4	2 (28.6%)	5 (71.4%)	
Smoking mother	NO	134 (61.5%)	84 (38.5%)	0.648
	YES	54 (58.7%)	38 (41.3%)	
Mother caries	NO	36 (70.6%)	15 (29.4%)	0.107
	YES	151 (58.5%)	107 (41.5%)	
Mother annual dental visits	0	9 (56.3%)	7 (43.8%)	0.966
	1	77 (59.7%)	52 (40.3%)	
	2	99 (61.5%)	62 (38.5%)	
	3	2 (66.7%)	1 (33.3%)	
Mother daily brushing	1	7 (70%)	3 (30%)	0.825
	2	112 (60.2%)	74 (39.8%)	
	3	67 60.4%)	44 (39.6%)	
Mother systemic fluoride during pregnancy	0	154 (61.1%)	98 (38.9%)	0.613
	1	31 (57.4%)	23 (42.6%)	
Smoking father	NO	115 (60.8%)	74 (39.2%)	0.645
	YES	61 (58.1%)	44 (41.9%)	
Father annual dental visits	0	22 (66.7%)	11 (33.3%)	0.39
	1	76 (62.8%)	45 (37.2%)	
	2	78 (56.1%)	61 (43.9%)	
Father caries	NO	40 (56.3%)	31 (43.7%)	0.486
	YES	136 (61%)	87 (39%)	

* Statistically significant variables.

## Data Availability

Data sharing are available at Bambino Gesù Children’s Hospital.

## Supplementary Materials

The following supporting information can be downloaded at: https://www.mdpi.com/article/10.3390/healthcare10040607/s1, Table S1: School questionnaire; Table S2: Dental clinical record.
