# Supplementary material for: Cross Sectional Study on the Association between Dental Caries and Life Habits in School Age Italian Children
Source: Healthcare (Basel). 2022 Mar 24;10(4):607. doi: 10.3390/healthcare10040607 (PMC9028244; doi:10.3390/healthcare10040607)
Supplement: Supplementary file 1 [file healthcare-10-00607-s001.zip › healthcare-1571657-supplementary.pdf]

Supplementary Materials

Table S1. School questionnaire.

Child personal information

Name	
Surname	
Date of birth	
Sex	
Place of Residence	

Mother's medical history

Born in Italy	☐ yes ☐ no
Place of Residence	
Weight	
Height	
Level of Education	☐ no education<br>☐ primary<br>☐ secondary<br>☐ high school graduate<br>☐ undergraduate<br>☐ postgraduate (master/doctorate)
Do you smoke?	☐ yes ☐ no
If YES, how many cigarettes a day?	
How many times a year do you visit your dentist?	☐ never<br>☐ one<br>☐ two<br>☐ more than two
Have you ever had dental caries on your permanent teeth?	☐ yes ☐ no
Have you ever extracted your teeth because of caries?	☐ yes ☐ no
When have you last visited your dentist?	☐ less than 6 months<br>☐ more than 6 months<br>☐ more than a year
How many times a day do you brush your teeth?	☐ never<br>☐ one<br>☐ two<br>☐ more than two
Did you take fluoride during pregnancy and/or feeding?	☐ yes ☐ no

Father's medical history

Born in Italy	□ yes □ no
Place of Residence	
Weight	
Height	
Level of Education	□ no education<br>□ primary<br>□ secondary<br>□ high school graduate<br>□ undergraduate<br>□ postgraduate (master/doctorate)
Do you smoke?	□ yes □ no
If YES, how many cigarettes a day?	
How many times a year do you visit your dentist?	□ never<br>□ one<br>□ two<br>□ more than two
Have you ever had dental caries on your permanent teeth?	□ yes □ no
Have you ever extracted your teeth because of caries?	□ yes □ no
When have you last visited your dentist?	□ less than 6 months<br>□ more than 6 months<br>□ more than a year
How many times a day do you brush your teeth?	□ never<br>□ one<br>□ two<br>□ more than two
How many members are there in your family?	□ 1 □ 2 □ 3 □ 4 □ 5 □ >5

Child's medical history

Was he/she delivered at term?	□ yes □ no
Was he/she breastfed?	□ yes □ no<br>How many months?
Was he/she fed with formula?	□ yes □ no<br>How many months?
Has the child ever taken fluoride?	□ yes □ no

If YES, up to what age?	
Have you ever been informed about your child's oral hygiene?	☐ yes ☐ no
How many times a day do you think you should brush your teeth?	☐ 1 ☐ 2 ☐ 3 ☐ >3
How many times a day does your child actually brush his/her teeth?	☐ 1 ☐ 2 ☐ 3 ☐ >3
Does he/she use a toothpaste with fluoride?	☐ yes ☐ no
Does he/she use dental floss?	☐ yes ☐ no
Does he/she use an electric toothbrush?	☐ yes ☐ no
Did he/she use a pacifier?	☐ yes ☐ no
If YES, up to what age?	
Do/did you usually put your child's pacifier in your mouth?	☐ yes ☐ no
Do/did you usually drink from your child's same bottle and/or glass?	☐ yes ☐ no
Do/did you usually use a honeyed pacifier for your child?	☐ yes ☐ no
Does/did your child usually drink or suck something else other than water before sleeping?	☐ yes ☐ no
Does/did your child use diuretics, mucolytics, cough suppressants, bronchodilators, sprays for asthma?	☐ yes ☐ no
Does your child often stay with his/her mouth open?	☐ yes ☐ no
Does your child suffer from allergies?	☐ yes ☐ no
If YES, to what?	
How many times a day does your child eat?	☐ 1 ☐ 2 ☐ 3 ☐ 4 ☐ 5 ☐ >5
Does he/she eat at night?	☐ yes ☐ no
When was his/her last dental visit?	☐ less than 6 months<br>☐ more than 6 months<br>☐ more than a year

Table S2. Dental clinical record.

Patient's Name and Surname			Date of Birth:
DENTAL GENERAL INFORMATION			
SEX	F	M	
AGE		Years, months	
WEIGHT		Kg	
HEIGHT		cm	
PRESENCE OF CARIES	YES	NO	
NUMBER OF DECAYED TEETH			
